# *Ehrlichia chaffeensis* Infections among HIV-infected Patients in Human Monocytic Ehrlichiosis–Endemic Area

**DOI:** 10.3201/eid0909.020560

**Published:** 2003-09

**Authors:** Thomas R. Talbot, James A. Comer, Karen C. Bloch

**Affiliations:** * Vanderbilt University School of Medicine, Nashville, Tennessee, USA; † Centers for Disease Control and Prevention, Atlanta, Georgia, USA

**Keywords:** Ehrlichiosis, *Ehrlichia chaffeensis*, HIV, epidemiology

## Abstract

Manifestations of human monocytic ehrlichiosis (HME), a tick-borne infection caused by *Ehrlichia chaffeensis*, range from asymptomatic disease to fulminant infection and may be particularly severe in persons infected with HIV. We conducted a serologic study to determine the epidemiology of HME in HIV-positive patients residing in an HME-endemic area. We reviewed charts from a cohort of 133 HIV-positive patients who were seen during the 1999 tick season with symptoms compatible with HME (n=36) or who were asymptomatic (n=97). When available, paired plasma samples obtained before and after the tick season were tested by using an indirect immunofluorescence assay (IFA) to detect antibodies reactive to *E. chaffeensis.* Two symptomatic incident cases were identified by IFA, resulting in a seroincidence of 6.67% among symptomatic HIV-positive participants with paired samples available for testing and 1.64% overall. The baseline seroprevalence of HME was 0%. In contrast to infection in immunocompetent patients, *E. chaffeensis* infection in HIV-positive persons typically causes symptomatic disease.

During the last 25 years, the discovery of a number of newly identified infectious agents, such as *Borrelia burgdorferi, Legionella pneumophila,* and HIV, has raised concern in both the medical and lay communities about novel infectious threats to human populations. Among these emerging pathogens are several species of *Ehrlichia*, small, gram-negative bacteria transmitted by arthropod vectors that can cause human disease, such as human monocytic ehrlichiosis (HME). First described in 1986 (*1*), HME is caused by *Ehrlichia chaffeensis*, an organism transmitted primarily by the lone star tick (*Amblyomma americanum*) (*2*). Infection with *E. chaffeensis* can range from being clinically asymptomatic to causing a severe life-threatening illness. HME typically causes systemic symptoms (including fever, headache, malaise, rash, abdominal pain, nausea, and cough) and laboratory signs (leukopenia, thrombocytopenia, and elevated transaminase levels). Rarely, patients have fulminant infection with disseminated intravascular coagulation, sepsis, and adult respiratory distress syndrome, leading to death (*2*). Asymptomatic infection with *E. chaffeensis* may occur frequently, as suggested in a recent seroepidemiologic study in which 67% of military recruits in an *E. chaffeensis*–endemic area seroconverted without symptoms (*3*).

The risk for HME in immunocompromised patients is unknown; however, numerous case reports and reviews have described severe ehrlichial infection in immunosuppressed patients (*4*–*6*), including several reports of rapidly fatal infection with *E. chaffeensis* in AIDS patients (*7*–*9*). Diagnosis of HME in HIV-positive patients is often confounded by the fact that the signs and symptoms of ehrlichial infection mimic typical findings commonly associated with HIV infection, its complications, and the medications commonly used in treating such patients. Delayed consideration and diagnosis of ehrlichial infection may result in additional illness if antibiotic therapy is not instituted promptly.

Studies investigating the epidemiology of *E. chaffeensis* infection have focused on healthy persons living in regions endemic for *E. chaffeensis* or clinical findings among hospitalized case-patients (*3*,*10*–*12*). A systematic evaluation of the seroepidemiology of ehrlichial disease in HIV-infected persons has not been performed. We therefore conducted a descriptive seroepidemiologic study to ascertain the prevalence and incidence of *E. chaffeensis* infections in HIV-infected persons located in an area endemic for HME.

## Methods

### Selection of Patients

Participants were selected among HIV-positive patients who receive their medical care at the Comprehensive Care Center, an adult HIV-oriented primary care clinic located in Nashville that serves middle Tennessee and surrounding regions. Center records were retrospectively analyzed to identify patients seen at the clinic for any reason between March 1, 1999, and October 31, 1999 (the typical period of tick activity in middle Tennessee).

### Symptomatic Patient Subset

Those patients discharged with diagnoses indicative of potential ehrlichial infection, according to the International Classification of Diseases, 9th Edition (ICD-9), were identified by means of a blinded database review. Specifically, patients who were assigned the following ICD-9 codes were selected for the study cohort: fever or fever of unknown origin (780.6), viral infection (079.9), upper respiratory infection (465.9) or respiratory disease (478.9) not otherwise specified, Lyme disease (088.81), rickettsial disease (specified, 083.8, or unspecified, 083.9), Rocky Mountain spotted fever (082.0), tick bite (088.89), and myalgias (729.1). To find potential participants who may have been missed in the original search, a second database search identified patients during the study period who were prescribed doxycycline, the therapy of choice in the empiric treatment of febrile illness during the tick season.

### Asymptomatic/Other Patient Subgroup

The rest of the study cohort comprised patients who visited the center during the study period and who had plasma banked for serologic investigation (see “Plasma Collection”). To investigate the incidence of asymptomatic *Ehrlichia* infection, patients were selected from a blinded review of the center’s plasma sample log. Patients who had banked plasma samples from the pretick season (between September 15, 1998, and March 31, 1999) as well as from the posttick season (after October 31, 1999) and who had visited the center for routine follow-up during the study period were selected for study. Records of patients identified as asymptomatic were reviewed for symptoms suggestive of HME during the study period that were not encoded with an ehrlichiosis-compatible ICD-9 diagnosis.

### Chart Review

The center’s data charts were analyzed for demographic data (age at start of study period, race, sex, number of clinic visits during the study period), past medical history, medication history (the administration of highly active antiretroviral therapy [HAART] and medication used as prophylaxis against opportunistic infections), and HIV status based on the most recent CD4 count and viral load drawn before the study period began (March 1, 1999). Charts from the symptomatic patients were further analyzed for symptoms suggestive of *Ehrlichia* infection, including the presence or absence of fever, headache, rash, fatigue, malaise, upper respiratory infection symptoms, nausea, vomiting, myalgias, abdominal pain, and mental status changes. Symptom history, laboratory parameters (peripheral leukocyte count, platelet count, aspartate aminotransferase, and alanine aminotransferase) at baseline and during the acute illness, and illness outcomes (including antibiotics prescribed, hospitalization, and death) were also collected. Insufficient data on tick exposure, tick bites, or outdoor activity were available to evaluate exposure risk factors for *Ehrlichia* infection.

### Plasma Collection

Since 1998, the center has maintained a repository of plasma samples frozen at –70°C by retaining specimens obtained from patients during routine phlebotomy. All patients who choose to participate in the plasma banking provide written informed consent based on a protocol approved by the Vanderbilt University Institutional Review Board. The plasma log was cross-checked with the study participant list identified from the database review as outlined above. Participants with no banked plasma sample from before the onset of the study period (preseason sample) or at the onset of clinical symptoms (acute sample) were excluded. Samples from at least 4 weeks after the acute clinical illness or after the study period (postseason sample) were also identified for most persons. Persons with no further samples banked after their acute illness or the study period were included only in determination of seroprevalence.

### Serologic Testing

All preseason and postseason samples were tested in a blinded fashion by indirect immunofluorescence assay for antibody reactive with *E. chaffeensis* with an assay previously described for human granulocytic ehrlichiosis, which has been widely employed for HME using different antigen substrates (*13*). A reciprocal antibody titer of >64 was considered elevated and indicative of infection with *E. chaffeensis*. Seroconversion to *E. chaffeensis* was defined as a fourfold or greater increase in antibody titer between acute-phase or preseason and convalescent-phase samples.

### Statistical Analysis

Incidence rates were described as the number of cases of seroconversion divided by the total population of interest. We used 95% confidence intervals determined by using Stata statistical software version 7.0 (Stata Corporation, College Station, TX).

## Results

We initially identified a total of 176 patients from the center’s records; 43 were excluded because specimens for testing were unavailable, leaving 133 in our study cohort. Thirty-six (27.1%) had symptoms compatible with HME (29 found by screening of ICD-9 codes and for doxycycline use, 7 found after chart review of initial asymptomatic candidates), and 97 (72.9%) had no symptoms suggestive of this diagnosis. Characteristics of the cohort are shown in the Table. The median CD4 count was 370 cells/mm^3^. Symptomatic participants had significantly more visits (p<0.001) to the clinic during the study period and were significantly (p=0.035) more likely to have received antibiotic therapy (excluding doxycycline; data not shown). As doxycycline was used to select for symptomatic participants, doxycycline therapy was, not included in the analysis of antibiotic use. Other characteristics (specifically age, gender, baseline CD4 count, baseline viral load, use of HAART, use of prophylaxis for opportunistic infection [*Pneumocystis carinii* pneumonia and *Mycobacterium avium* complex], and average number of visits during the study period) between the symptomatic and asymptomatic subgroups did not differ significantly.

**Table:** Baseline characteristics of study cohort of HIV-positive persons residing in Tennessee^a^

Characteristic	N (% or range)
Age (mean, y)	38.8 (21–75)
Sex	
Male	107 (80.5%)
Female	26 (19.5%)
Baseline CD4 count (median, cells/mm^3^)	370 (6–1,200)
Baseline viral load (median, copies/dL)	1,003 (<400–>750,000)
On prophylaxis	
HAART	122 (91.7%)
OI prophylaxis	70 (52.6%)
PCP prophylaxis^b^	66 (49.6%)
MAC prophylaxis^c^	31 (23.3%)
Average number of clinic visits^d^	4.75 (1–13)
Treated with antibiotic therapy^d,e^	40/133 (30.1%)
Treated with doxycycline^d^	14/133 (10.5%)
Hospitalized^d^	7/133 (5.3%)

^a^HAART, highly active antiretroviral therapy; OI, opportunistic infection; PCP, *Pneumocystis carinii* pneumonia; MAC, *Mycobacterium avium* complex.           ^b^PCP prophylaxis: use of trimethoprim-sulfamethoxazole, dapsone, or aerosolized pentamidine therapy.           ^c^MAC prophylaxis: use of azithromycin or clarithromycin therapy.           ^d^During the study period.           ^e^Antibiotics used to treat the ongoing clinical symptoms; persons taking antibiotic therapy specifically for OI prophylaxis alone were not included.

None of the patient specimens obtained before the 1999 tick season had serologic evidence of prior *Ehrlichia* infection, resulting in a baseline seroprevalence of 0% for our cohort. Of the 122 patients with paired samples available (92 asymptomatic, 30 symptomatic), 1 patient had a clinical syndrome compatible with HME and had a significant rise in antibody titer to *E. chaffeensis* during the study period (initial titer 64; postseason titer 1,024). Clinically notable disease characterized by fever, myalgias/arthralgias, leukopenia, and thrombocytopenia developed in this patient after tick exposure and required a 4-day hospitalization. During this hospitalization, his diagnosis was confirmed by conducting a polymerase chain reaction assay on his serum, which was positive for *E. chaffeensis*. His symptoms resolved after a course of doxycycline. A second patient with a 10-day history of symptoms compatible with HME (fatigue, cough, and overall malaise), but no documentation of tick exposure or tick bite, had an initial acute-phase titer of 512, drawn when first seen by a clinician (10 days after symptom onset), fulfilling case criteria for probable *Ehrlichia* infection (*14*). This patient did not have an earlier preseason sample available for analysis but did have a postseason titer of 512 obtained 4 months after clinical illness, suggesting a prolonged elevation in antibody titer. He was thought to have an upper respiratory tract infection by his primary caregiver, and doxycycline was prescribed for his illness. His symptoms resolved without hospitalization.

These two cases resulted in a seroincidence among symptomatic patients of 6.67% (95% confidence interval [CI] 0.82, 22.1) and an overall incidence of 1.64% (95% CI 0.2, 5.8). No asymptomatic cases were identified in our cohort (upper 95% CI for seroconversion in the asymptomatic population, 3.2%).

## Discussion

Researchers have conducted various serologic studies to ascertain the epidemiology of *E. chaffeensis* infection in specific populations. Carpenter et al. (*10*) found a seroincidence of 25.7% in febrile patients in North Carolina with a history of a recent tick bite. In a prospective seroepidemiologic study of residents living in a rural community in California, prevalence rates of 4.6% were reported, and most of the infected participants recalled no recent compatible illness (*11*). In a comparison of two golf-oriented retirement communities in middle Tennessee, one abutting a wildlife-management area and one 20 miles away from the area used as a control population, Standaert et al. found seroprevalence rates of 12.5% and 3.3%, respectively (*12*). A study on the seroprevalence in children residing in HME-endemic areas, including Tennessee, found a seroprevalence rate (as defined by *E. chaffeensis* antibody titer >1:80) of nearly 15% among children undergoing phlebotomy in Nashville (*15*). None of these studies, however, investigated the incidence rates for immunosuppressed persons, such as persons infected with HIV, who may be at increased risk for symptomatic disease after ehrlichial infection.

Our findings indicate that the prevalence and incidence of HME attributable to *E. chaffeensis* infection in an HIV-positive population are quite low in a cohort of HIV-positive patients receiving care at an urban HIV clinic within an HME-endemic region. The incidence rate in our study was similar to those previously reported in a cohort of healthy military recruits in an area endemic for *E. chaffeensis* (1.3%) (*3*). However, only 33.3% of seropositive persons in that study had a compatible febrile illness, and none of these symptomatic seroconverters were sufficiently ill to require medical care (*3*). In contrast, both of our case-patients had symptomatic disease of sufficient severity to require medical care, and one required hospitalization. Furthermore, none of our patients had serologic evidence of asymptomatic infection during the study period. Therefore, while the overall incidence of *Ehrlichia* infection was not increased in our cohort, these results are in agreement with other studies that indicate that HME can cause severe infection in HIV-positive persons.

Our patient with a diagnosis of probable HME had evidence of a sustained antibody response. The acute-phase serum sample and a convalescent-phase sample obtained 4 months later both had titers of 512. This finding suggests that the immune response mounted by HIV-positive persons against *E. chaffeensis* is durable and may persist for several months, similar to the response seen in HIV-negative persons. Because of the low rate of seroconversion in our cohort, we were unable to analyze data on specific risk factors (e.g., CD4 count or use of HAART) that might predispose persons with HIV infection to ehrlichiosis.

Our study had several limitations. The retrospective design placed constraints on the data that could be abstracted, thus introducing possible reporting bias. A prospective study, in contrast, would allow investigators to collect further information on exposure risks, such as level of outdoor activity, and could reduce the variability in symptom reporting found with our study. Our study population could also lead to bias and, as a result, limit generalizability of our results to the HIV-positive population as a whole. The Comprehensive Care Center draws patients from both metropolitan areas (Nashville) and rural communities; however, our cohort may have been more metropolitan and less likely to come into contact with wooded environments. Also, HIV-infected patients who regularly attended the clinic may have had more contact with the healthcare delivery system and thus been more likely to take regular antiretroviral medications that could reduce their viral burden and concomitant immunodeficiency. As a result, those patients who are noncompliant with follow-up (and, by extension, antiviral therapy) may be at greater risk for symptomatic infection and may have been missed in our analysis.

The use of serologic methods to determine actual prevalence and incidence rates for the HIV-infected population may also be problematic. A reduced antibody response to various antigens, including those contained in tetanus and pneumococcal vaccines, in HIV-infected patients has been described in previous studies (*16*). A potentially decreased ability to mount an immune response to *E. chaffeensis* may have led to false-negative antibody titers and an underestimate of the incidence of ehrlichiosis in this population. Such a finding was highlighted in two previous reports of HIV-positive persons with fatal *E. chaffeensis* infection who did not mount an antibody response during their illnesses (*5*,*7*). The serologic response to ehrlichial infection may also be blunted or inhibited by tetracycline therapy, which, when given early in the course of *Ehrlichia* infections, inhibits the development of a serologic response (*10*,*12*). Empiric treatment of febrile patients with a clinical picture resembling ehrlichiosis in our population thus could have blunted the antibody response and led to a falsely low seroincidence and prevalence.

In conclusion, we found that levels of HME infection in our HIV-positive cohort were similar to those in normal, healthy persons who received intense exposure to the outdoors in an HME-endemic area. However, both of our case-patients had clinical infections, one requiring hospitalization. Caregivers of HIV-positive patients in regions endemic for *E. chaffeensis* should consider ehrlichiosis as part of the growing list of potential opportunistic infections and maintain a high level of clinical suspicion for this disease. Prospective studies in HIV-positive populations are needed to fully understand the extent of infection with *E. chaffeensis* in these patients.
